# Atropine in Acute Asthma

**Published:** 1909-05-29

**Authors:** 


### ATROPINE IN ACUTE ASTHMA.

The treatments that have been recommended for asthma at different times are very numerous—sprays, medicines, diets, cauterisations, inhalations, and so on—and no two authorities are in absolute agreement as to the best prophylactic treatment between the attacks.

There are many who hold that atropine is the ideal medicine for the relief of the acute attack itself. Stimulation of the vagus in the cat causes spasmodic contraction of the muscles of the bronchioles precisely similar to that which occurs reflexly or spontaneously in asthma. Pressure on the vagus in man is capable of calling forth an attack of asthma; a tumour irritating that nerve has been known to do so. Experiments show that atropine paralyses the nerve endings of the vagus in the bronchioles. Hence it is easy to explain scientifically why it is that the subcutaneous injection of $\frac{1}{150}$ to $\frac{1}{100}$ grain of atropine stops the stormiest symptoms of an attack of asthma in from ten to twelve minutes. The effect of the single dose may pass off before the attack of asthma is itself exhausted, and, therefore, to prolong the good effects of the atropine it is often wise to give morphine at the same time, in a dose of not less than $\frac{1}{8}$ grain.

Various celebrated sprays and inhalations that undoubtedly give great relief in asthma cases contain atropine as one of their chief ingredients. Ritsert's inhalation fluid contains atropine, belladonna, stramonium, and saltpetre.

Two useful formulæ which may conveniently be prescribed as inhalations, or altered to suit individual cases, are as follow:—

| | | |
|---|---|---|
| (1) | Atropine nitrite | 0.6 |
| | Cocaine nitrite. | 1.0 |
| | Glycerin | 32.0 |
| | Water | to 100 parts, and |
| (2) | Atropine sulphate | 0.15 |
| | Sodium nitrite | 0.6 |
| | Glycerine | 2 |
| | Water | 15 parts. |

Atropine may also be used as a prophylactic against an asthma attack. Trousseau's method is to prescribe $\frac{1}{150}$ grain of atropine in pill form daily for two days, then increasing the dose by $\frac{1}{150}$ grain in the day every second day until the patient takes altogether $\frac{1}{50}$ grain three times a day. This is continued for four weeks, and in many cases the results are good.

